# Antibodies from women urogenitally infected with *C. trachomatis *predominantly recognized the plasmid protein pgp3 in a conformation-dependent manner

**DOI:** 10.1186/1471-2180-8-90

**Published:** 2008-06-09

**Authors:** Zhongyu Li, Youmin Zhong, Lei Lei, Yimou Wu, Shiping Wang, Guangming Zhong

**Affiliations:** 1 Department of Microbiology and Immunology, University of Texas Health Science Center at San Antonio, 7703 Floyd Curl Drive, San Antonio, TX 78229, USA; 2 Department of Parasitology, Xiangya Medical School, The Central South University, 168 Tongzipo Rd., Changsha, Hunan 410078, PR China; 3 Department of Microbiology and Immunology, University of South China, Changsheng West Rd, Hengyang, Hunan 421001, PR China

## Abstract

**Background:**

*C. trachomatis *organisms carry a cryptic plasmid that encodes 8 open reading frames designated as pORF1 to 8. It is not clear whether all 8 pORFs are expressed during *C. trachomatis *infection in humans and information on the functionality of the plasmid proteins is also very limited.

**Results:**

When antibodies from women urogenitally infected with *C. trachomatis *were reacted with the plasmid proteins, all 8 pORFs were positively recognized by one or more human antibody samples with the recognition of pORF5 protein (known as pgp3) by most antibodies and with the highest titers. The antibody recognition of the pORFs was blocked by *C. trachomatis*-infected HeLa but not normal HeLa cell lysates. The pgp3 fusion protein-purified human IgG detected the endogenous pgp3 in the cytosol of *C. trachomatis*-infected cells with an intracellular distribution pattern similar to that of CPAF, a chlamydial genome-encoded protease factor. However, the human antibodies no longer recognized pgp3 but maintained recognition of CPAF when both antigens were linearized or heat-denatured. The pgp3 conformation is likely maintained by the C-terminal 75% amino acid sequence since further deletion blocked the binding by the human antibodies and two conformation-dependent mouse monoclonal antibodies.

**Conclusion:**

The plasmid-encoded 8 proteins are both expressed and immunogenic with pgp3 as the most immunodominant antigen during chlamydial infection in humans. More importantly, the human anti-pgp3 antibodies are highly conformation-dependent. These observations have provided important information for further understanding the function of the plasmid-encoded proteins and exploring the utility of pgp3 in chlamydial diagnosis and vaccination.

## Background

*C. trachomatis*, consisting of many different serovars ranging from A to L plus various subtypes, with serovars A to C mainly infecting human ocular epithelial tissues, potentially leading to preventable blindness [1], and D to K infecting human urogenital tracts, which can potentially cause severe complications such as ectopic pregnancy and infertility [2]. The L or LGV (lymphogranuloma venereum) organisms including serovars L1–3 are more invasive than other urogenital tract serovars and can also infect rectal tissues. The L2 organisms recently caused several outbreaks in certain human populations [3,4]. MoPn (mouse pneumonitis agent) used to be classified as a murine biovar of *C. trachomatis *is now categorized as an independent species called *C. muridarum *despite the high degree of genome sequence conservation between MoPn and *C. trachomatis *serovars. Nevertheless, MoPn has been extensively used in a mouse urogenital infection model to study *C. trachomatis *pathogenesis and immune responses [5-7]. Despite the apparent differences in tissue tropism, all *C. trachomatis *serovars including MoPn undergo a common intracellular biphasic growth cycle [8]. A typical infection starts with the entry of elementary bodies (EBs), the infectious form, into host cells via endocytosis [9]. The internalized EBs can rapidly differentiate into reticulate bodies (RBs), the metabolically active but non-infectious form of chlamydial organisms. After numerous rounds of replication, the RBs can differentiate back into EBs prior to spreading to adjacent cells. All Chlamydia species can accomplish its entire biosynthesis, replication and differentiation within the cytoplasmic vacuole (also termed inclusion). The successful intracellular replication along with the infection-induced inflammatory responses is thought to be mainly responsible for Chlamydia-induced diseases [10].

Besides a highly conserved genome, all *C. trachomatis *serovars also contain a 7.5 kb cryptic plasmid [11]. The plasmids from serovars A (pCTA; ref: [12], B (pCTT1; ref: [13], D (pCHL1; ref: [14], L1 (pLGV440; ref: [15], L2 (pLGV2; ref: [16] and MoPn Nigg strain (pMoPn; ref: [11,17] have been sequenced. The plasmid sequences are very similar (>96% amino acid sequence identity between different *C. trachomatis *human serovars and 82% between MoPn and the *C. trachomatis *human serovars), all coding for 8 putative ORFs designated as pORF1 to 8 [11]. The wide distribution of the cryptic plasmid suggests that there is a positive selection for maintaining the plasmids to benefit chlamydial survival. At the same time, chlamydial strains/isolates that are either deficient in the plasmid or carry mutated plasmids have been identified [18-23], suggesting that there might also be host immune selection pressure against the plasmid-encoded antigens and the plasmid-encoded function can be compensated by genes/proteins encoded elsewhere. To understand the functions of the plasmid-encoded proteins, we tested whether the plasmid proteins are expressed and immunogenic during *C. trachomatis *infection in humans in the current study. Since it is difficult to directly detect chlamydial proteins and evaluate chlamydial protein immunogenicity in humans, we detected the recognition of chlamydial fusion proteins by human antibodies in ELISA as an indirect indicator for both chlamydial protein expression and immunogenicity in individuals with *C. trachomatis *infection. We found that the plasmid-encoded 8 proteins were recognized by one or more human serum samples, suggesting that they were all made during human infection. Importantly, we found that pORF5 (pgp3) was the most immunodominant antigen among the 8 plasmid proteins and as dominant as CPAF, a chlamydial genome-encoded protease factor known to be immunodominant and secrete into host cell cytosol. Indeed, the pgp3 fusion protein-purified human IgG detected the endogenous pgp3 in the cytosol of *C. trachomatis*-infected cells in addition to its intra-inclusion localization. Interestingly, the human antibody recognition of pgp3 but not CPAF as highly conformation-dependent since linearizing or denaturing either pgp3 fusion protein or the endogenous protein blocked the human antibody recognition of pgp3 while similar treatments to CPAF still permitted a significant recognition of CPAF by the same human antibodies. These observations have not only demonstrated that the fusion protein ELISA is a relevant experimental system for analyzing antibody responses to chlamydial infection in humans, but also more importantly, provided useful information for further developing pgp3 as a diagnostic reagent and/or vaccine candidate.

## Results

### 1. Human antibody recognition of C. trachomatis plasmid proteins

**Figure 1:**
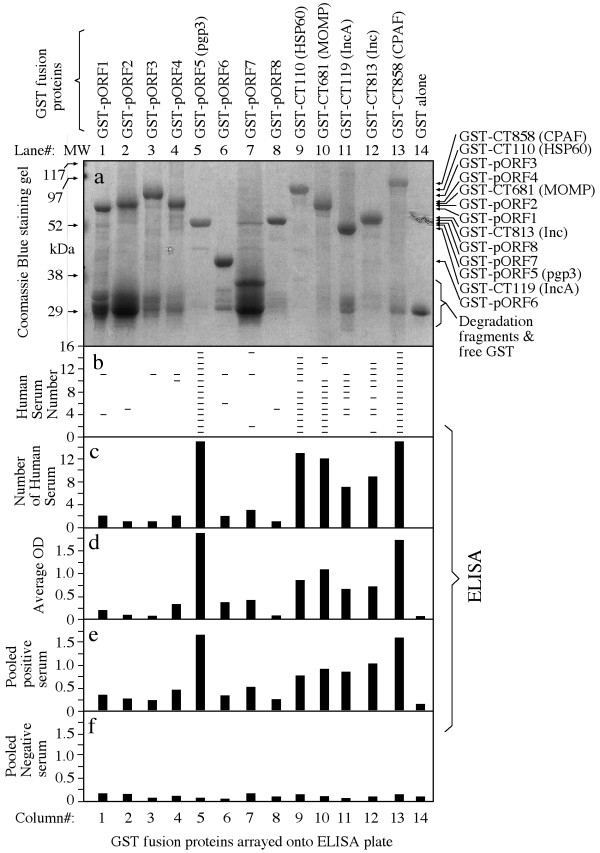
**Reactivity of human antibodies with chlamydial fusion proteins**. The GST fusion proteins were precipitated with glutathione-agarose bead from bacterial lysates and the precipitates were checked in SDS polyacrylamide gel for quality (panel a). The same lysates were used as the source of fusion proteins in ELISA. 15 patient serum antibodies (along the Y-axis of panel b) were each diluted at 1:500 and reacted with the bacterial lysates containing GST-chlamydial fusion proteins or GST alone (listed both on the top and in the bottom of the figure). Each positive reaction was marked with a horizontal bar (panel b) and the number of sera reacted with each fusion protein was plotted in panel c. The average ODs were calculated (panel d). The 15 sera were pooled and measured against the fusion proteins (panel e). A pooled serum from 8 health individuals (pooled negative serum) was used to similarly react with the fusion proteins (panel f). Note that pgp3 and CPAF fusion proteins were recognized by most antiesra and with the highest titers.

**Figure 2:**
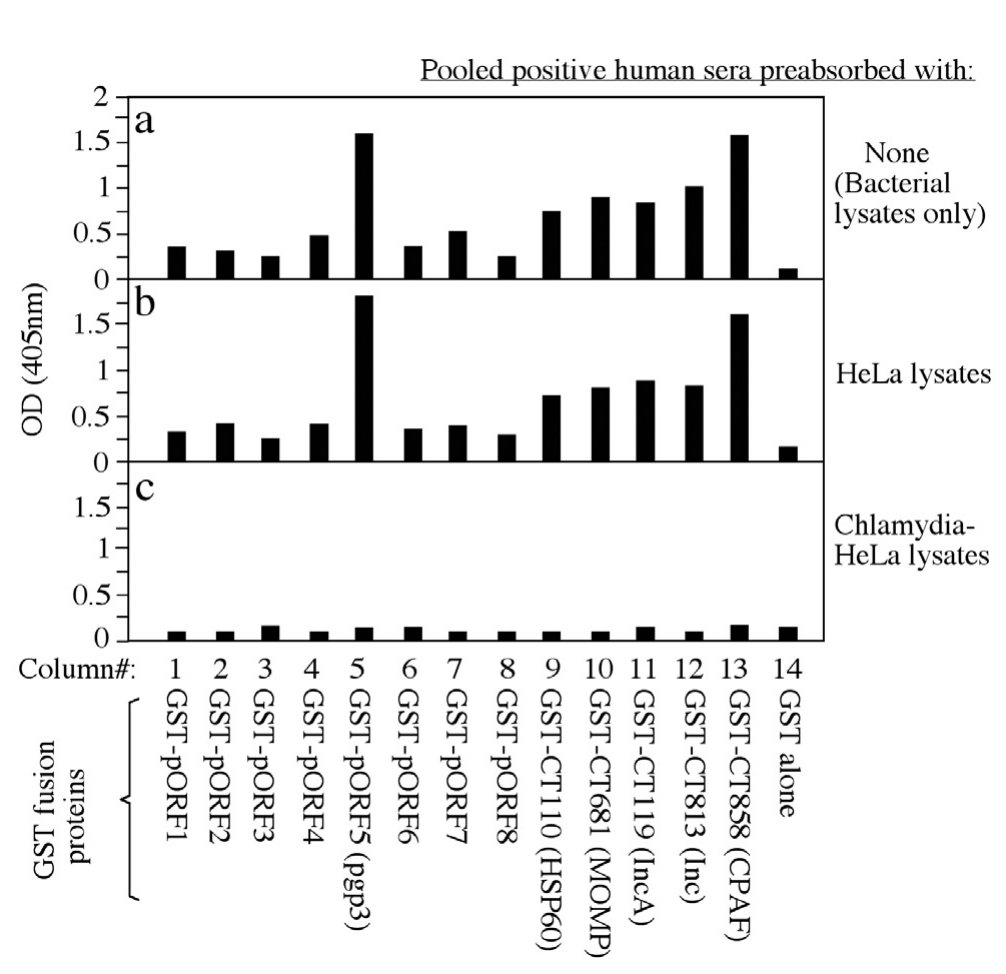
**The effect of preabsorption with chlamydial antigens on human antibody reactivity with the fusion proteins**. The pooled positive human serum from 15 patients was absorbed with or without HeLa alone or serovar D-infected HeLa cell lysates (as indicated along the right side of the figure) prior to reacting with the fusion proteins listed in the bottom of the figure in ELISA. The pooled positive serum reactivity with chlamydial fusion proteins was completely blocked by the absorption with the *C. trachomatis*-infected HeLa cell lysates (panel c) but not HeLa alone lysates (panel b).

**Figure 3:**
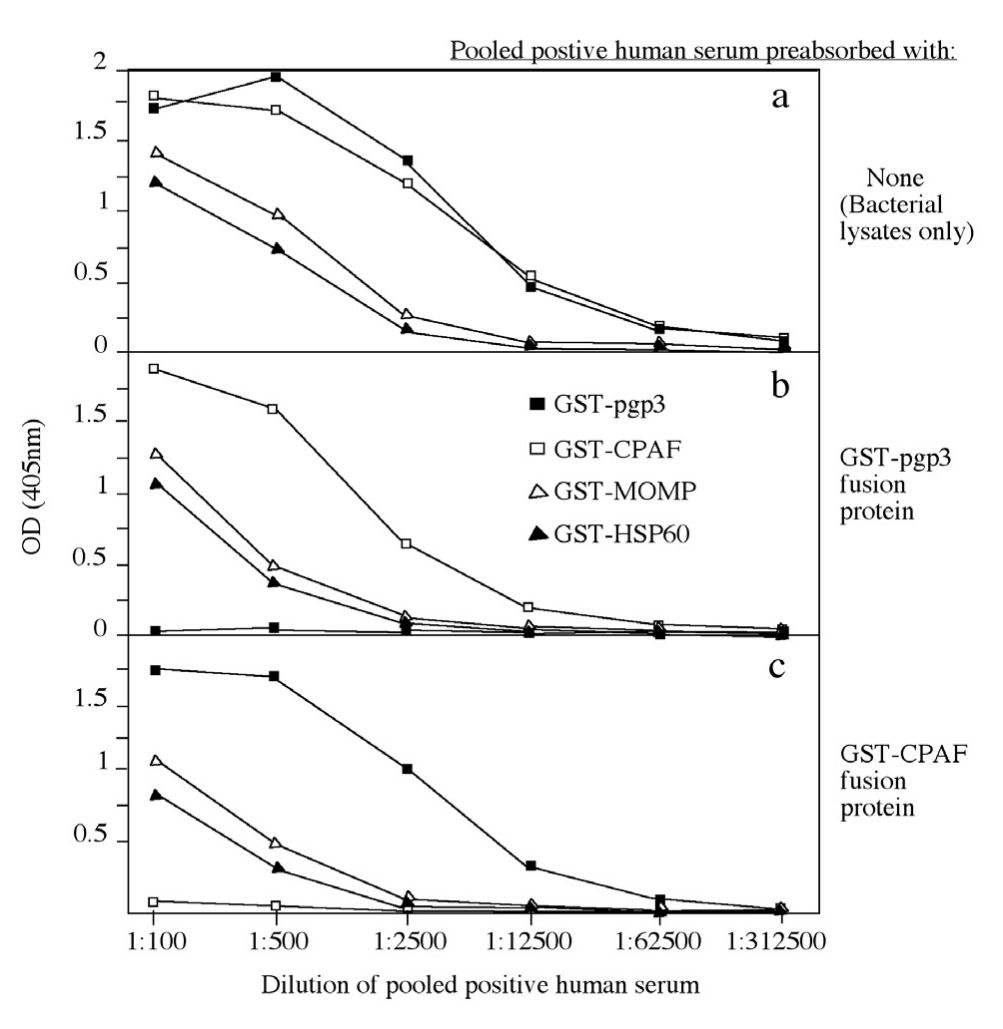
**The effect of preabsorption with GST fusion proteins on human antibody reactivity with the microplate-immobilized fusion proteins**. The pooled human serum was 5-fold serially diluted as indicated along the X-axis and reacted with the microplate-immobilized GST-pgp3 (filled square), GST-CPAF (open square), GST-MOMP (open triangle) and GST-HSP60 (filled triangle). The reactivity was expressed as OD at 405 nm as indicated along the Y-axis. The pre-absorption of the pooled antiserum with GST-pgp3 fusion protein only blocked the human antibody binding to the pgp3 but not other fusion proteins (panel b) while the pre-absorption with GST-CPAF fusion protein only blocked antibody binding to CPAF but not other fusion proteins (panel c).

**Figure 4:**
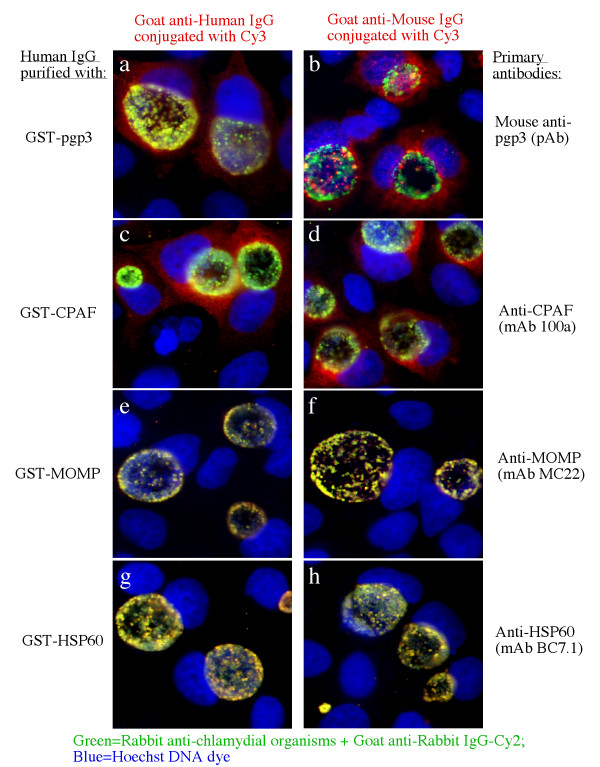
**Detection of endogenous chlamydial antigens in *C. trachomatis *serovar L2-infected cells by fusion protein-purified human IgG (panels a, c, e & g) or antigen-specific mouse antibodies (panels b, d, f & h)**. The primary antibody staining was visualized with either a goat anti-human IgG Cy3 or goat anti-mouse IgG Cy3 conjugates (red). The chlamydial inclusions were visualized with a rabbit anti-chlamydial organism antibody plus a goat anti-rabbit IgG Cy2 conjugate (green). The DNA was visualized by the Hoechst DNA dye (blue). Note that both the pgp3 and CPAF-purified human IgG localized the corresponding endogenous antigens inside the host cell cytosol of the *C. trachomatis*-infected cells.

To determine whether the plasmid-encoded proteins are expressed and immunogenic during *C. trachomatis *infection in humans, the 8 pORFs were expressed as GST fusion proteins and the fusion proteins were reacted with 15 human antisera in an ELISA (Fig. 1). Each of the 8 plasmid fusion proteins were positively recognized by at least one antiserum (panel b, column# 1 to 8), suggesting that the plasmid proteins are all expressed during human infection. However, there is a great variation in both the antibody binding frequency and titer among the plasmid proteins. The plasmid protein pORF5 (also called pgp3) was recognized by all fifteen human antisera, pORF7 by three, pORF1, 4 & 6 by two and pORF2, 3 & 8 by one only. Five *C. trachomatis *genome-encoded proteins were used as immunodominant antigen controls and each of them was recognized by 7 or more human antiserum samples (column# 9-13), which is consistent with our previous observations [24,25]. Surprisingly, the plasmid-encoded pgp3 was as immunodominant as CPAF, a chlamydial secreted protease factor known to be most immunodominant when evaluated together with many other *C. trachomatis *genome-encoded proteins [25] and to induce protective immunity against chlamydial diseases when tested in a murine urogenital infection model [5]. The immunodominance was not only reflected by the high frequency of human antiserum recognition (panel c, both pgp3 and CPAF were positively recognized by all 15 human antisera) but also the high antibody binding titers. Both pgp3 and CPAF displayed the highest antibody binding titers when the titers of each individual antiserum binding to the fusion proteins were averaged (panel d) or when the 15 antiserum samples were pooled at equal ratio and assayed against the fusion proteins (e). A pooled negative antiserum failed to react with any of the fusion proteins significantly (panel f). To further confirm the antibody binding specificity, we used the lysates made from either *C. trachomatis*-infected HeLa cells or HeLa cells alone to pre-absorb the human antisera prior to reacting with the fusion proteins in ELISA (Fig. 2). We found that the reactivity of the pooled positive antiserum with the fusion proteins (panel a) was completely removed by the absorption with the *C. trachomatis*-infected cell lysates (panel c) but not HeLa alone lysates (panel b), demonstrating that the human antibodies reactive with the fusion proteins were also able to recognize the chlamydial endogenous antigens produced during infection in HeLa cells. The above observations together have suggested that all 8 plasmid proteins are expressed by *C. trachomatis *during human infection and pgp3 is most immunogenic. Since both pgp3 and CPAF were dominantly recognized by the human antibodies and the antibody reactivity with both was blocked by the *C. trachomatis*-infected cell lysates, we further evaluated whether there was a cross-reactivity between pgp3 and CPAF by human antibodies (Fig. 3). We found that preabsorption of the pooled human positive antiserum with pgp3 fusion proteins only blocked the antibody reactivity with pgp3 but not CPAF, MOMP or HSP60 (panel b) while preabsorption with CPAF fusion protein blocked the antibody binding to CPAF but not pgp3, MOMP or HSP60 (panel c), demonstrating that the pgp3-reactive human antibodies did not cross-react with CPAF or vice versa. Interestingly, the human antibodies purified with pgp3 or CPAF fusion proteins both detected the endogenous proteins in the cytosol of the *C. trachomatis*-infected cells in addition to their intra-inclusion localization (Fig. 4, panels a & c) while the MOMP or HSP60 fusion protein-purified human antibodies detected signals only inside the inclusions (panels e & f). The localization of the corresponding endogenous chlamydial proteins was confirmed with antigen-specific mouse antibodies (panels b, d, f & h).

### 2. Mapping pgp3 immunodominant regions

**Figure 5:**
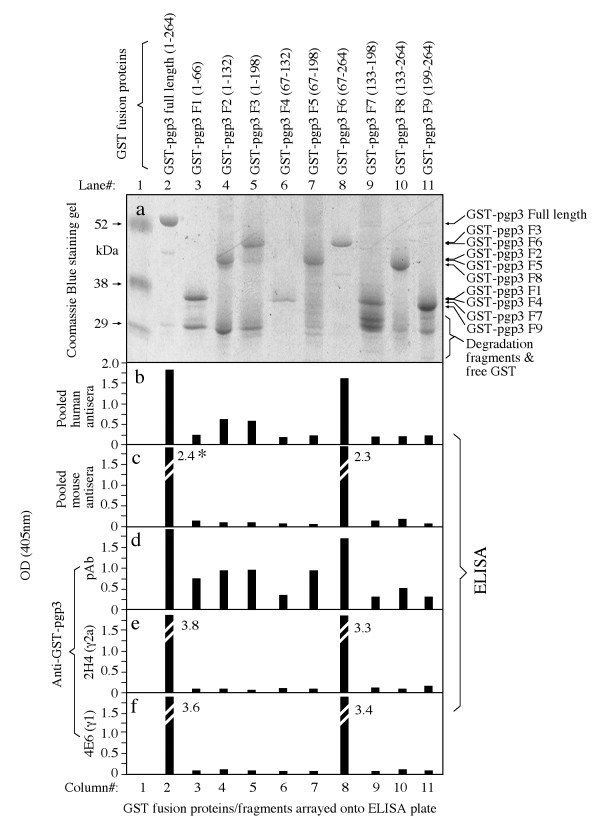
**Mapping immunodominant regions of pgp3 by reacting the full-length pgp3 and nine fragment GST fusion proteins (panel a) with the pooled human antisera (b) and mouse sera (c) respectively**. The polyclonal antibody (pAb, d) and mAbs (clone 2H4, e; clone 4E6, f) raised by immunizing mice with pgp3 fusion protein. The antibody reactivity was displayed as OD along the Y-axis. "*" the numbers in each panel indicate the actual OD values obtained for a particular antigen-antibody interaction as marked.

**Figure 6:**
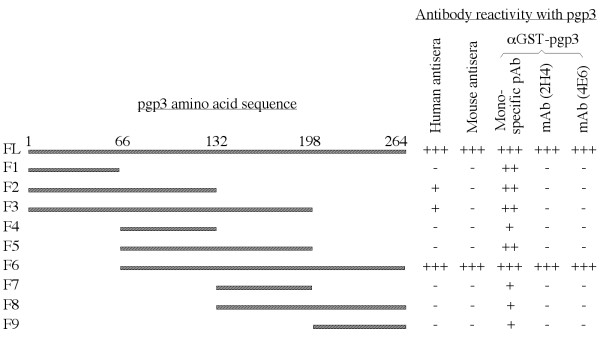
**A summary of the reactivity between the GST fusion proteins and various antibodies measured in Fig. 5**. "+" stands for minimal, "++" reasonable recognition and "+++" strong reactivity. Note that the two mAbs and the pooled mouse or human sera only dominantly recognized the full length pgp3 and the fragment 6 (missing the N-terminal 66 amino acids) while the pgp3-immunized mouse antibody recognized pgp3 and its many fragments.

The robust recognition of pgp3 by human antibodies motivated us to further identify the pgp3 regions responsible for the immunodominance. The pgp3 protein consisting of 264 amino acids was expressed in 9 different fragments with each varying by 66 amino acids (designated as F1 to 9, Fig. 6, left side) in the form of GST fusion proteins (Fig. 5, panel a). When these 9 fragments along with the full length pgp3 fusion proteins were reacted with the pooled positive human antiserum sample, we found that the F6 fragment lacking the N-terminal 66 amino acids was recognized by the human antibodies as strongly as the whole pgp3 protein was. However, no other fragments were significantly recognized although there was a minimal reactivity of F2 & 3 with the human antibodies (Fig. 5, panel b). The sera pooled from mice urogenitally infected with live chlamydial organisms also strictly recognized the full-length pgp3 and fragment 6 (panel c). However, the antiserum raised by immunizing mice with pgp3 fusion protein recognized all fragments, suggesting that all fragments can be immunogenic if presented to host immune cells. Nevertheless, the highest reactivity of the pgp3 immunized mouse serum was still with the full-length and F6 (panel d), suggesting that many antibody species produced during pgp3 fusion protein immunization mimicked the specificities of human or mouse antibodies produced during live infection. Indeed, two monoclonal antibodies (mAbs) were selected out from the pgp3 fusion protein-immunized mice and both only recognized the full-length pgp3 and fragment 6 (panels e-f). These observations have demonstrated that the C-terminal three quarters of pgp3 amino acid sequence is required for maintaining a conformation that is recognizable by human and mouse anti-pgp3 antibodies.

### 3. Human antibody recognition of pgp3 is highly conformation-dependant

**Figure 7:**
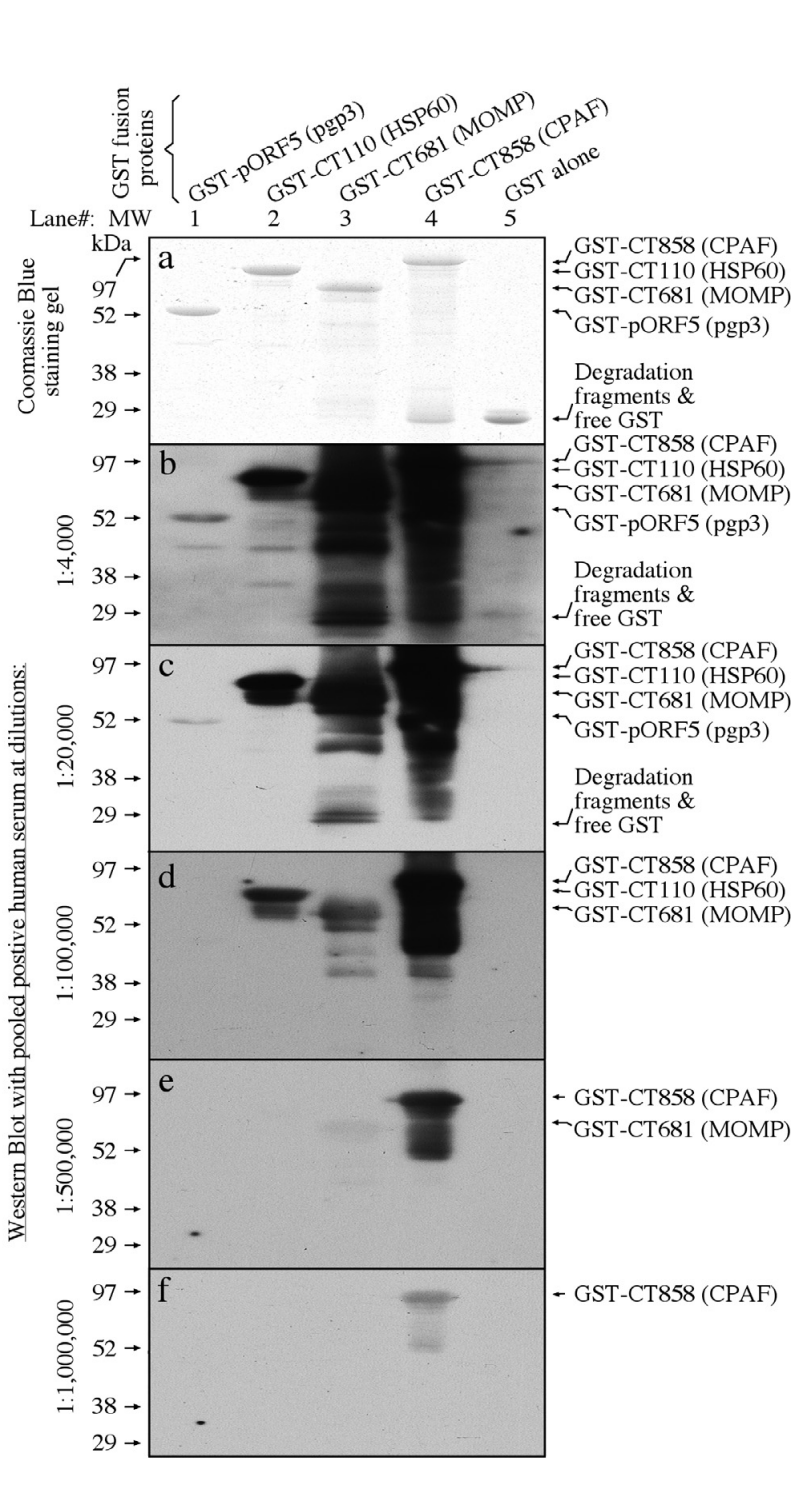
**Reactivity of human antibodies with pgp3 and control fusion proteins on Western blot**. (A) GST fusion proteins as listed on top of the figure were analyzed in SDS gel (panel a) and parallel gels were blotted onto nitrocellulose membrane for reacting with the pooled positive human antiserum at various dilutions as listed along the left of the figure. The primary antibody reactivity was visualized with a goat anti-human IgG conjugated with HRP in ECL as described in the method section. The corresponding protein bands were indicated on the right of the figure. Note that the pooled human antiserum only minimally reacted with the pgp3 band at 1:4,000 (panel b) while remaining reactive with CPAF even after 1:1,000,000 dilution (panel f), suggesting that the human anti-pgp3 antibodies are highly conformation-dependent.

**Figure 8:**
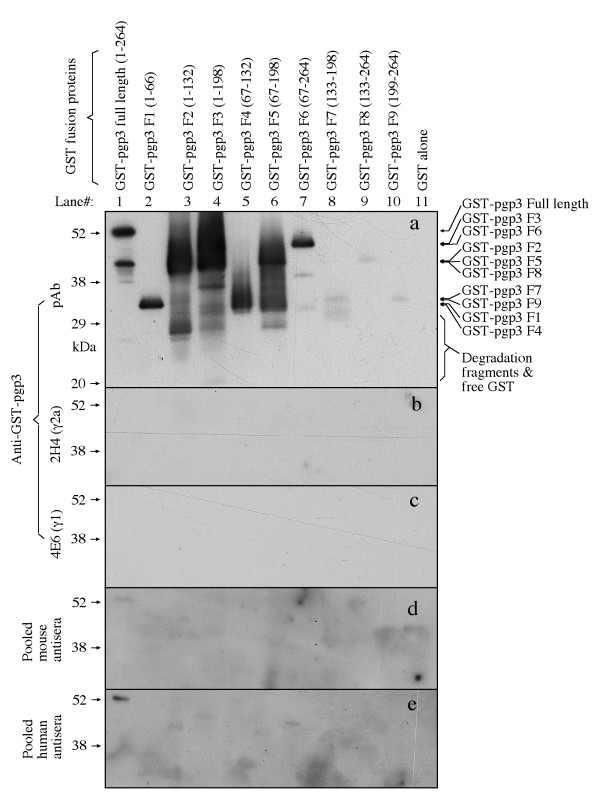
**Reactivity of human antibodies with pgp3 fragment fusion proteins**. The pgp3 and its fragment fusion proteins were reacted with the pooled human at 1:4,000 dilution (panel e) and mouse antisera at 1:4,000 (d), both produced during live chlamydial infection, and polyclonal antibody (pAb, a) and mAbs (clone 2H4, b; clone 4E6, c) raised by immunizing mice with pgp3 fusion protein as listed along the left of the figure. The corresponding protein bands were indicated on the right of the figure. Note that only the anti-pgp3 fusion protein-raised antibody recognized pgp3 and its many fragments and no other antibodies picked any significant signals.

When we attempted to use Western blot to confirm the human antibody binding to the chlamydial fusion proteins, we found that pgp3 was no longer recognizable by the human antibodies (Fig. 7). Although the human antibodies recognized both pgp3 and CPAF fusion proteins with an equivalent titer in ELISA (Fig. 3), the same antibody sample only minimally recognized the pgp3 at 1:4000 (Fig. 7, panel b) while was still able to bind CPAF even after 1:1000,000 dilution (panel f) in a Western blot assay. Clearly, linearizing fusion proteins in SDS gel dramatically reduced the human antibody binding to pgp3. However, the linearized pgp3 full length or fragment fusion proteins were significantly recognized by the mouse antibody raised by immunizing mice with GST-pgp3 (Fig. 8, panel a), suggesting that pgp3 sequences were antigenic even after linearization and the lack of recognition of the linearized pgp3 by the human antibody was due to lack of the appropriate antibody specificities. The observations that the linearized pgp3 and its fragment fusion proteins were not recognized by antisera produced during live chlamydial infection either in human (Fig. 8, panel e) or mice (d) or the pgp3-specific mAbs (b & c) while all these antibodies recognized pgp3 in ELISA (Fig. 5) have demonstrated that these antibodies are conformation-dependent.

**Figure 9:**
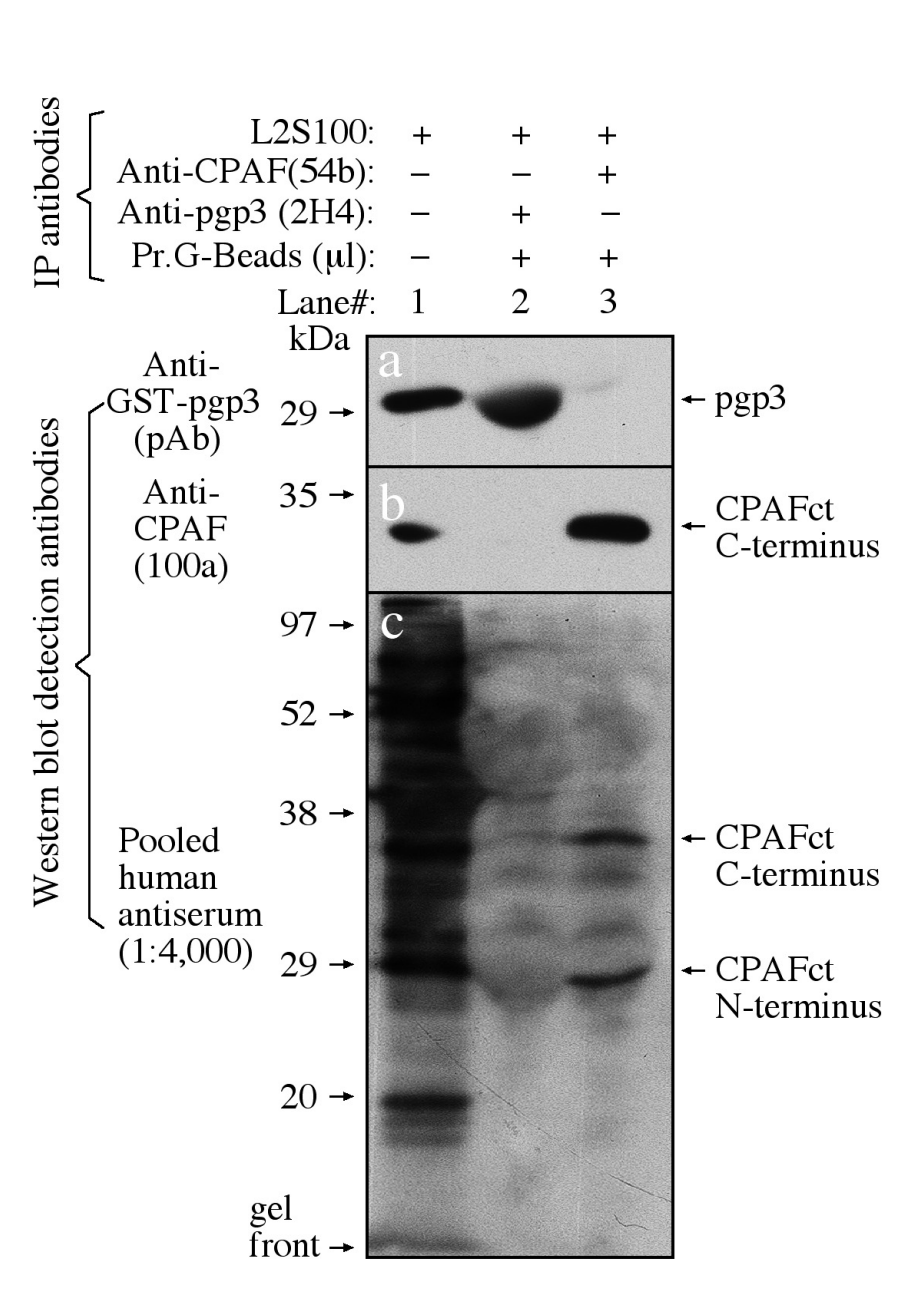
**Reactivity of human antibodies with endogenous pgp3 on Western blot**. The *C. trachomatis *L2-infected cell cytosolic preps (L2S100) were precipitated with protein G agarose beads (Pr.G-Beads) bound with anti-pgp3 (mAb clone 2H4) or anti-CPAF (clone 54b) antibodies as indicated on top of the figure. The precipitates were resolved in SDS gels and blotted for reacting with the anti-GSP-pgp3 (pAb; panel a), anti-CPAF (100a; b) or the pooled positive human antiserum (c) in a Western blot as listed along the left of the figure. Lane#1 was loaded with the L2S100 without precipitation as positive antigen control. The corresponding protein bands were indicated on the right of the figure. Note that human antibodies detected both the precipitated endogenous CPAF C- & N-terminal fragments but not pgp3.

**Figure 10:**
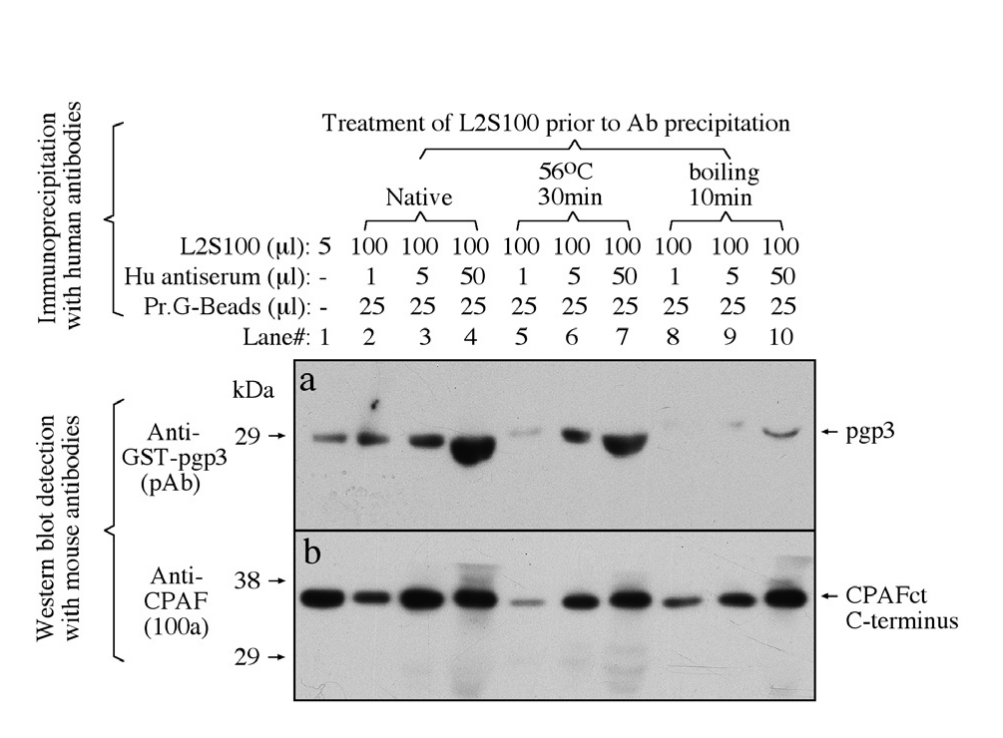
**Reactivity of human antibodies with endogenous pgp3 in immunoprecipitation**. Various amounts of the pooled positive human antiserum was used to precipitate chlamydial endogenous antigens from L2S100 with or without prior heat treatments either at 56°C for 30 min or boiling for 10 min. The human antibody precipitates were detected with either the anti-pgp3 polyclonal (pAb; panel a) or anti-CPAF monoclonal (clone 100a, panel b) antibodies as shown on the left of the figure. The corresponding protein bands were indicated on the right of the figure. Note that although the human antibodies precipitated both the endogenous pgp3 and CPAF from the native L2S100, these human antibodies no longer reacted with pgp3 in boiled L2S100 sample but maintaining the ability to pull down CPAF.

We next tested whether the human antibody recognition of the endogenous pgp3 was also conformation-dependent. When the endogenous pgp3 and CPAF were precipitated with specific antibodies and used as the antigens in a Western blot (Fig. 9), we found that the human antibody only recognized CPAF (panel c, lane 3) but not pgp3 (lane 2), demonstrating that the endogenous pgp3 after linearization was not recognizable by the human antibodies, confirming the results obtained with fusion proteins. We further compared the effects of heat treatment of antigens on human antibody recognition of pgp3 and CPAF (Fig. 10). The human antibodies successfully precipitated down both pgp3 and CPAF from the *C. trachomatis*-infected cell cytosolic samples (lane#2-4). However, heat treatment of the cytosolic samples by boiling for 10 min significantly blocked the human antibody precipitation of pgp3 but not CPAF, suggesting that most pgp3-specific antibody species in the human antisera recognized pgp3 epitopes that are heat-labile.

## Discussion

Although the *C*. *trachomatis *plasmid is predicted to encode 8 putative ORFs, it is not known whether these proteins are expressed and immunogenic during chlamydial infection in humans. Here, we have used a fusion protein ELISA approach to analyze human antibody responses to *C. trachomatis *infection and demonstrated that all the 8 pORFs are both expressed and immunogenic during chlamydial human infection. Mre importantly, we have presented convincing evidence that pORF5 or pgp3 is a most immunodominant antigen and antibodies produced against this protein during live chlamydial infection are highly conformation-dependent. First, the 15 antisera from women urogenitally infected with *C. trachomatis *all recognized pgp3 with high titers while the rest 7 pORF fusion proteins were recognized by the human antisera at much lower frequencies and titers. Second, by comparing to other known immunodominant antigens encoded in the *C. trachomatis *genome, including the traditionally known strong antigens HSP60 and MOMP [26,27] and the recently discovered immundominant antigens IncA, CT813 (Inc; ref: [24] and CPAF [25,28], pgp3 was recognized by the human antibodies as dominantly as CPAF, a most immunodominant antigen among all chlamydial proteins analyzed so far [25]. Finally, although both pgp3 and CPAF are immunodominant in women with *C. trachomatis *urogenital infection, pgp3 but not CPAF recognition by human antibodies was blocked by either linearization or boiling of the antigens, demonstrating the conformation-dependence of the human anti-pgp3 antibodies. The finding on the restricted conformational dependence of human antibody recognition of pgp3 was a surprise to us. It is generally thought that epitopes of membrane proteins such as MOMP are highly conformation-dependent. However, significant amounts of human antibodies still recognized MOMP on Western blot while only a minimal recognition of pgp3 was detected in the same assay, suggesting that pgp3 can stably maintain its conformation to elicit conformation-dependent antibodies during infection in humans.

Although both human and animal antibodies have previously been shown to react with the plasmid-encoded 28 kDa pgp3, the results varied a lot. While some reported more than 80% patients who were positive for *C. trachomatis*-specific antibodies on MIF (micro-immunofluorescence assay) reacted with pgp3 [29], other reports indicated a much lower detection rate of the anti-pgp3 antibodies in *C. trachomatis*-infected individuals with a detection frequency as low as 57–59% [30,31]. It appeared that a higher detection rate was achieved when the pgp3 antigen was used in soluble form [32] while antibodies from Chlamydia-infected animals or humans (even at a dilution as low as 1:100) could only detect a week signal of pgp3 on Western blot [33]. These varied detection results led to the hypothesis that the human anti-pgp3 antibodies may be conformation-dependent [32,34]. However, no serious effort was made to test the hypothesis. The current study has comprehensively compared the antibody recognition of pgp3 and CPAF under various native and denaturing conditions and presented the first compelling experimental evidence demonstrating that anti-pgp3 antibodies produced during chlamydial live infection are indeed highly conformation-dependant.

Interestingly, pgp3 immunization via a DNA vaccination induced a protective immunity that significantly reduced the shedding of live organisms after an intra-vaginal challenge infection with *C. trachomatis *serovar D [35]. Coincidentally, immunization with CPAF, another secreted chlamydial protein that was dominantly recognized by human antibodies, also induced protective immunity against chlamydial infection [5]. Furthermore, the CPAF-induced immunity even reduced pathologies in mouse oviducts induced by chlamydial urogenital challenge infection. It will be interesting to evaluate whether the pgp3 immunization can also decrease the Chlamydia-induced pathologies in the mouse oviducts. Given the new knowledge that pgp3 is a highly conformation-dependent antigen, whether immunization with native-like pgp3 protein can induce more relevant immunity against chlamydial infection deserves further evaluation. This hypothesis is worth testing since pgp3 also localizes inside the inclusion and may even be a component of the outer membrane complex [34].

## Methods

### 1. Chlamydial infection

The *C. trachoamtis *serovars D, L2 and MoPn (*C. muridarum *Nigg strain) organisms were propagated in HeLa cells (human cervical carcinoma epithelial cells, ATCC cat# CCL2), purified, aliquoted and stored as described previously [24]. To infect HeLa cells, cells grown in either 24 well plates with coverslips or tissue flasks containing DMEM (GIBCO BRL, Rockville, MD) with 10% fetal calf serum (FCS; GIBCO BRL) at 37°C in an incubator supplied with 5% CO_2 _were inoculated with chlamydial organisms (servers D or L2) at an MOI of 0.5 (or as indicated in individual experiments) as described previously [24]. The infected cultures were processed at different time points after infection for either inmmunofluorescence assays or Western blot analyses as described below. To infect mice (Balb/c, female, 6–8 week old, JAX^® ^Mice and Services, Bar Harbor, Maine 04609), the MoPn organisms were intra-vaginally inoculated into mice at a dose of 5 × 10^4 ^IFUs (inclusion forming units) per mouse as described previously [5]. The infection was monitored by quantitating the IFUs recovered from the mouse vaginal swabs. Eighty days post infection, mouse blood was collected for preparing the mouse antisera. The serum samples from 5 mice were pooled together at an equal ratio, designated as the pooled mouse antiserum.

### 2. Fusion protein production and fusion protein ELISA

The eight pORFs encoded by the pCHL1 plasmid [14] from *C. trachomatis *serovar D organisms were cloned into pGEX vectors (Amersham Pharmacia Biotech, Inc., Piscataway, NJ). The cloned pORFs were expressed as fusion proteins with glutathione-s-transferase (GST) fused to the N-terminus of the chlamydial proteins as previously described [25]. The fusion proteins were purified using glutathione-conjugated agarose beads (Pharmacia) and the purified proteins were used to immunize mice for producing both polyclonal antibodies (pAb; ref: [36] and monoclonal antibodies (mAb; ref: [26,37]. Two mAbs were successfully produced against pORF5 (pgp3), designated as clones 2H4 (IgG2a) and 4E6 (IgG1). In addition, pgp3 was also expressed in 9 different fragments designated as F1 to F9 for the purpose of mapping immunodominant regions recognized by human or mouse antibodies. The primers for cloning the nine pgp3 fragments are as follows: F1 forward primer 5'*CGC*-GGATCC (restriction site)-ATG GGA AAT TCT GGT TTT TAT TTG (overlapping region)-3', reverse 5'*TTTTCCTTTT*-GCGGCCGC-TTA AGA AGC ATT GGT TGA TGA ATT-3'; F2 forward primer is the same as F1 forward, reverse 5'*TTTTCCTTTT*-GCGGCCGC-TTA GTT GCA TTG AAT TTT ATT AGT G-3'; F3 forward primer is the same as F1 forward, reverse 5'*TTTTCCTTTT*-GCGGCCGC-TTA TGA GTA TCC ATA ACT AAT CG-3'; F4 forward primer 5'-*CGC*-GGATCC-ATT ACA ATT GGT TTG GTA GCG G-3', reverse 5'-*TTTTCCTTTT*-GCGGCCGC-TTA GTT GCA TTG AAT TTT ATT AGT G-3';F5 forward primer is the same as F4 forward, reverse 5'-*TTTTCCTTTT*-GCGGCCGC-TTA TGA GTA TCC ATA ACT AAT CG-3'; F6 forward primer is the same as F4 forward, reverse 5'-*TTTTCCTTTT*-GCGGCCGC-TTA AGC GTT TGT TTG AGG TAT TA-3'; F7 forward primer 5'-*CGC*-GGATCC-GGG TTA TTC ACT CCC AGT AAC-3', reverse 5'-*TTTTCCTTTT*-GCGGCCGC-TTA TGA GTA TCC ATA ACT AAT CG-3'; F8 forward primer is the same as F7 forward, reverse 5'-*TTTTCCTTTT*-GCGGCCGC-TTA AGC GTT TGT TTG AGG TAT TA-3'; F9 forward primer 5'-*CGC*-GGATCC-TCA GGC ATT CCT AAT TTA TGT AG-3'. The F9 reverse primer is the same as F8 reverse primer. The GST fusion proteins or GST alone were immobilized onto glutathione-coated microplates (Pierce, Rockford, IL) as antigens in the fusion protein ELISA as described previously [25]. Briefly, after the appropriate protein induction, the bacteria were harvested to make lysates and the lysates were aliquoted and stored at -80°C. The quality of the expressed fusion proteins was assessed by purifying the fusion proteins from a portion of the lysates using the glutathione-conjugated agarose beads (Amersham Biosciences Corp). The fusion proteins were checked on SDS-polyacrylamide gels stained with a Coomassie blue dye (Sigma). The bacterial lysates that showed a prominent band at the expected molecular weight position were used for the microplate ELISA.

Human serum samples were collected from women seen in the Project SAFE research clinic in San Antonio and diagnosed with *C. trachomatis *cervical infections. The diagnosis was based on the detection of *C. trachomatis*-specific nucleic acids in endocervical secretions using a ligase chain reaction method without distinguishing the serotypes of the organisms (Abbott LCX, Abbot Laboratories, Chicargo, IL). The sera were collected at the time of clinic visits and stored in aliquots at -20°C. An IRB exempt permit is in place for the current study. The results from 15 human antisera were presented in the current study. In some experiments, the 15 human antisera from *C. trachomatis*-infected individuals were also pooled at equal ratio for analyses and the pooled serum was designated as pooled positive human antiserum. A total of 8 sera from healthy female individuals without *C. trachomatis *infection were similarly pooled (pooled negative antiserum) and used as negative controls. To minimize the detection of cross-reactive antibodies (human sera may contain antibodies reactive with bacterial antigens that potentially contaminate the microplate wells during fusion protein array), all serum samples were pre-absorbed with bacterial lysates. The bacterial lysates were made in the same way as the fusion protein-containing lysates were made except that the XL1-blue bacteria transformed with the pGEX-6p-2 vector plasmid were used. Both the patient and health individual serum samples after the pre-absorption were titrated for their ability to recognize chlamydial antigens on an immunofluorescence assay. Although the patient sera displayed high antibody titers (>1:1,000) in recognizing chlamydial antigens, the normal sera did not show any significant binding to the chlamydial antigens (<1:20). For the microplate array assay, the pre-absorbed serum samples were diluted in PBS containing 10% FCS and applied to the fusion protein-bound microplates for 2 hrs at RT. After washing, HRP (Horse Radish Peroxidase)-conjugated goat anti-human IgG (Jackson ImmunoResearch Laboratories, Inc., West Grove, PA) in combination with substrate ABTS (Sigma) was used to visualize the primary antibody binding. The human antibody binding to chlamydial fusion proteins was quantitated by reading the absorbance (OD) at 405 nm in a microplate reader (Molecular device, Ramsey, MN). In some assays, the human antibody samples were further absorbed with lysates made from either HeLa cells alone or *C. trachomatis *serovar D-infected HeLa cells at 4°C overnight in addition to the bacterial lysate absorption.

### 3. Immunofluorescence assay

HeLa cells grown on coverslips were fixed with 2% paraformaldehyde (Sigma, St. Luis, MO) dissolved in PBS for 30 min at room temperature, followed by permeabilization with 1% saponin (Sigma) for an additional 30 min. After washing and blocking, the cell samples were subjected to antibody and chemical staining. Hoechst (blue, Sigma) was used to visualize nuclear DNA. A rabbit anti-chlamydial organism antibody (R1L2, raised with *C. trachomatis *L2 organisms, unpublished data) plus a goat anti-rabbit IgG secondary antibody conjugated with Cy2 (green; Jackson ImmunoResearch Laboratories, Inc., West Grove, PA) was used to visualize chlamydial inclusions. The various mouse antibodies plus a goat anti-mouse IgG conjugated with Cy3 (red; Jackson ImmunoResearch) were used to visualize the corresponding antigens. The mouse antibodies include: pAbs made against the pORF5(pgp3)-GST fusion proteins (current study) and mAbs 100a against CPAFct C-terminus [36], MC22 against the major outer membrane protein (MOMP) and BC7.1 against chlamydial HSP60. In some cases, the primary antibodies were human IgG molecules purified with the corresponding GST fusion proteins-conjugated to glutathione-agarose beads. To visualize the binding of the purified human IgG antibodies to chlamydial antigens, a goat anti-human IgG conjugated with Cy3 (red; Jackson ImmunoResearch Laboratories, Inc.) was used.

The cell samples after the appropriate immuno-labeling were used for image analysis and acquisition with an Olympus AX-70 fluorescence microscope equipped with multiple filter sets (Olympus, Melville, NY) as described previously [36,38]. Briefly, the multi-color-labeled samples were exposed under a given filter set at a time and the single color images were acquired using a Hamamatsu digital camera. The single color images were then superimposed with the software SimplePCI to display multi-colors. An Olympus FluoView™ Laser Confocal Microscope (Olympus) was used to further analyze the co-stained samples at the UTHSCSA institutional core facility as described previously [39,40]. All microscopic images were processed using the Adobe Photoshop program (Adobe Systems, San Jose, CA).

### 4. Western blot assay

The Western blot assay was carried out as described elsewhere [36,41,42]. Briefly, the purified fusion protein, Chlamydia-infected cell cytosolic fraction or antibody-precipitated endogenous chlamydial protein samples were solublized in 2% SDS sample buffer and loaded into SDS polyacrylamide gel wells. The cytosolic fraction also called S100 was prepared as previously described [36,43]. In some cases, cytosolic preps from Chlamydia-infected cells (L2S100) were precipitated with protein G agarose beads (Pharmacia) bound with human antibodies, anti-pgp3 (mAb clone 2H4) or anti-CPAF (clone 54b) antibodies and the precipitates were resolved in SDS gels for Western blot. After electrophoresis, the resolved protein bands were transferred to nitrocellulose membranes and the membrane blots were detected with primary antibodies, including the pooled positive or negative human antisera, mouse pAbs produced during chlamydial live infection in mice or raised with GST-pgp3 fusion protein via immunization of mice or mouse mAbs clone 100a against CPAF C-terminus [36], clones 2H4 & 4E6 against pgp3. The primary antibody binding was probed with an HRP (horse radish peroxidase)-conjugated goat anti-human or mouse IgG secondary antibody (Jackson Immunologicals, Westgrove, PA) and visualized using the enhanced chemiluminescence (ECL) kit (Santa Cruz Biotech).

## Competing interests

The authors declare that they have no competing interests.

## Authors' contributions

All authors participated in the design of the study. ZL carried out most of the experiments and worked out a lot of technique challenges. YZ & LL expressed some of the plasmid proteins and produced antibodies. GZ conceived of the study and YW & SW participated in its design and coordination.
